# Supplementary material for: Adjacent single-atom irons boosting molecular oxygen activation on MnO2
Source: Nat Commun. 2021 Sep 14;12:5422. doi: 10.1038/s41467-021-25726-w (PMC8440510; doi:10.1038/s41467-021-25726-w)
Supplement: Supplementary file 1 — Supplementary Information [file 41467_2021_25726_MOESM1_ESM.pdf]

Supplementary Information

Adjacent single-atom irons boosting molecular oxygen activation on MnO_2

Huayu Gu, Xiao Liu*, Xiufan Liu, Cancan Ling, Kai Wei, Guangming Zhan, Yanbing Guo and Lizhi Zhang*

H. Gu, Prof. X. Liu, X. Liu, C. Ling, K. Wei, G. Zhan, Prof. Y. Guo, Prof. L. Zhang
Key Laboratory of Pesticide & Chemical Biology of Ministry of Education, College of Chemistry,
Central China Normal University, Wuhan 430079, P. R. China
E-mail: liuxiao71@mail.ccnu.edu.cn; zhanglz@mail.ccnu.edu.cn

Supplementary Figures

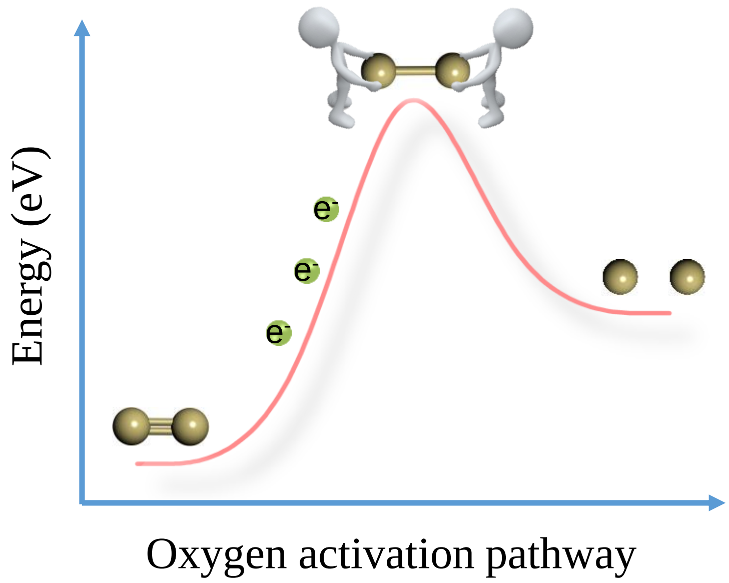

Supplementary Figure 1. Diagram of molecular oxygen activation.

Molecular oxygen activation is a continuous process of adsorption and dissociation of O_2 on the catalyst surface. The process relies on the transfer of electrons from the surface of catalyst to the O_2 to weaken the oxygen-oxygen double bond [1,2]. Meanwhile, the structure of the adsorption site on the catalyst surface plays an important role in the activation of O_2.

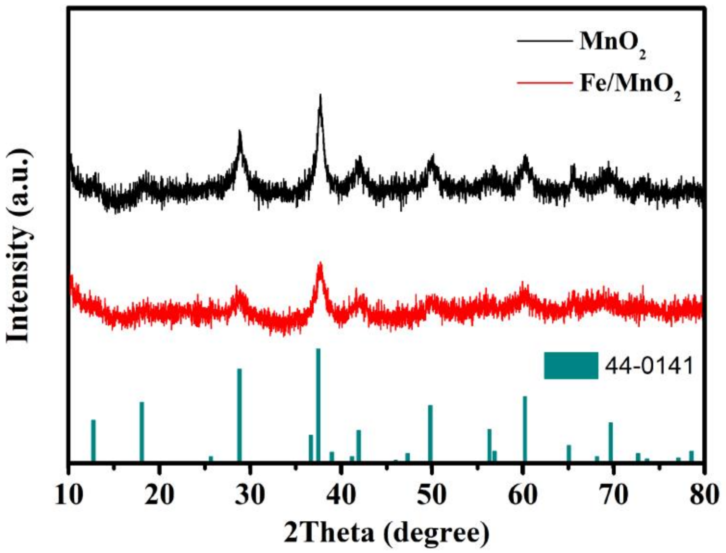

Supplementary Figure 2. XRD patterns of MnO_2 and Fe/MnO_2 (0.25% theoretical Fe content) as well as the standard card of MnO_2.

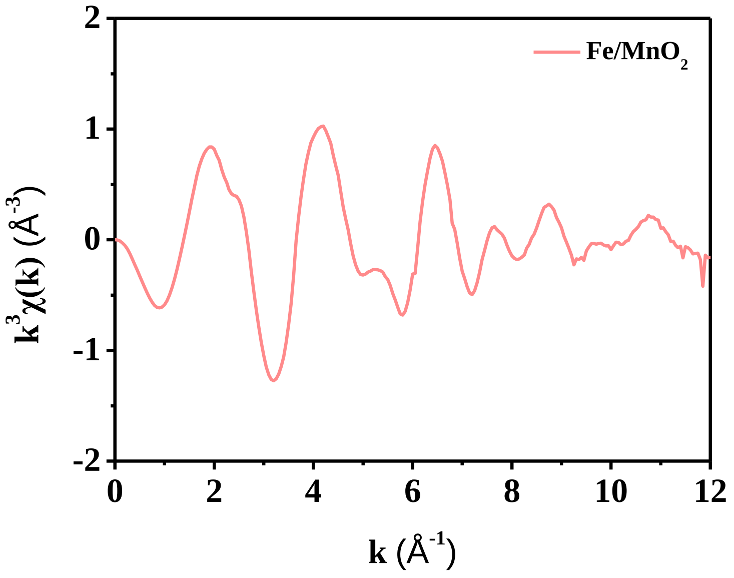

Supplementary Figure 3. k3-weighted EXAFS spectrum of Fe_2O_3 sample.

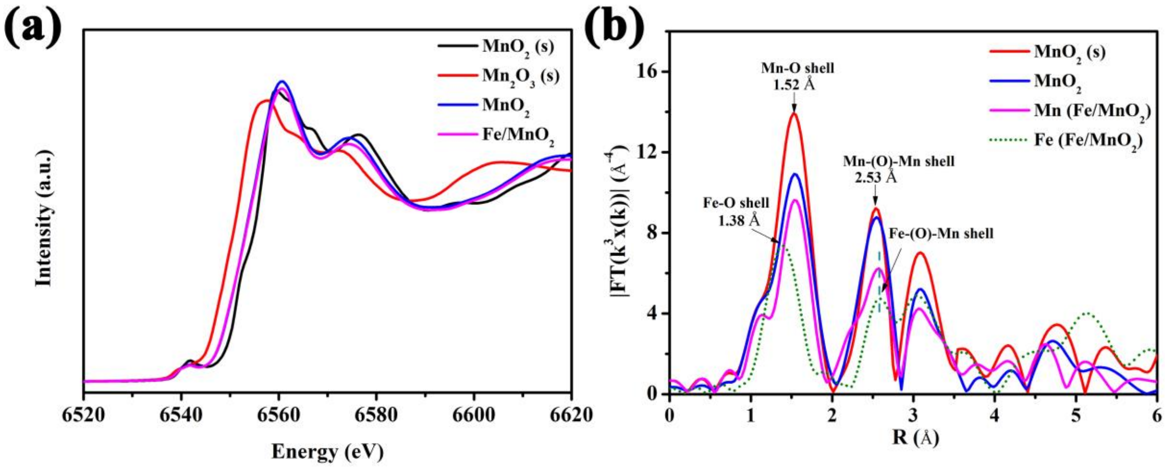

Supplementary Figure 4. The normalized XANES spectra (a) and Fourier-transformed K-edge EXAFS spectra in R-space (b) of Mn (without phase correction). MnO_2 (s), Mn_2O_3 (s) represent standard reagents in spectra.

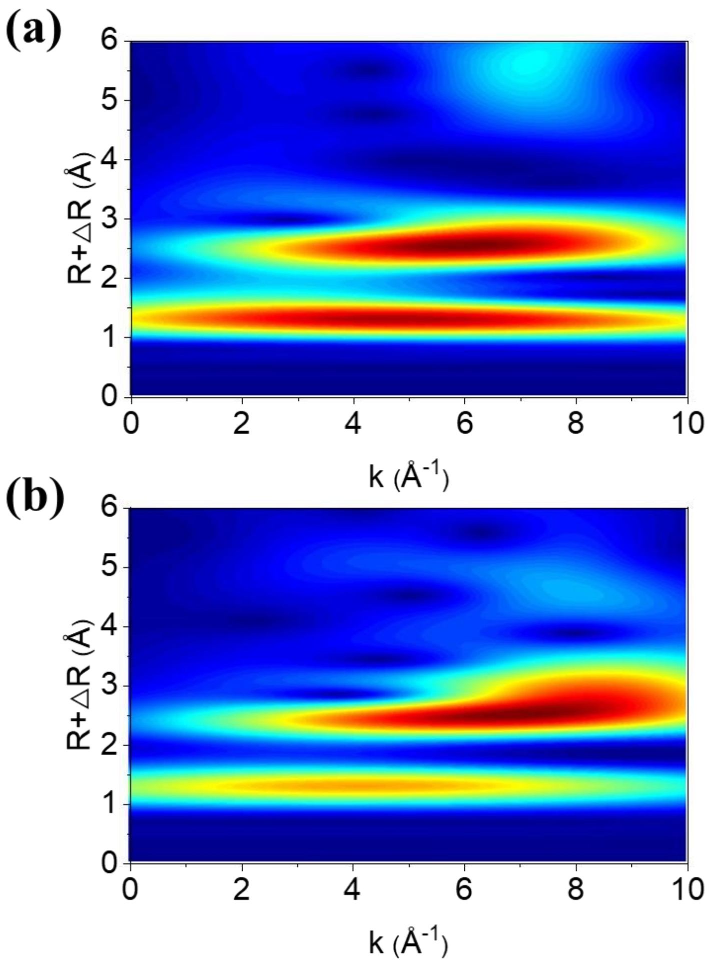

Supplementary Figure 5. Morlet wavelet transform for (a) Fe/MnO_2 and (b) Fe_2O_3.

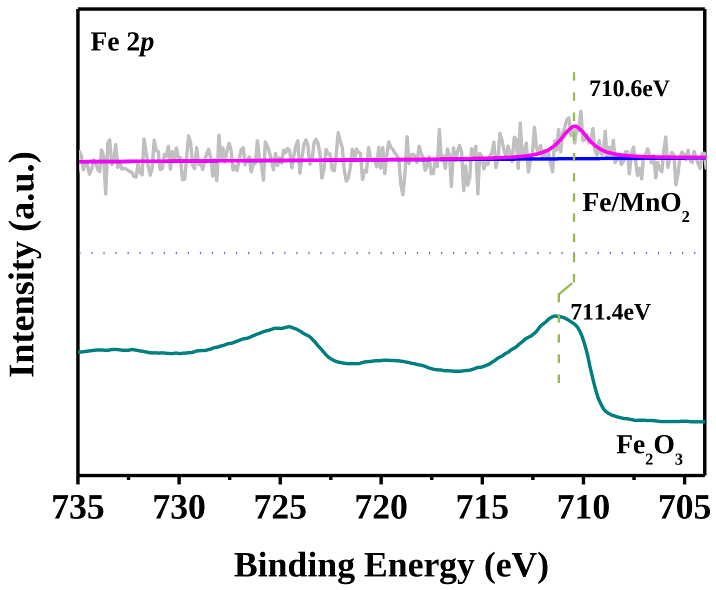

Supplementary Figure 6. XPS spectra of Fe 2*p* in Fe/MnO_2 and Fe_2O_3 sample.

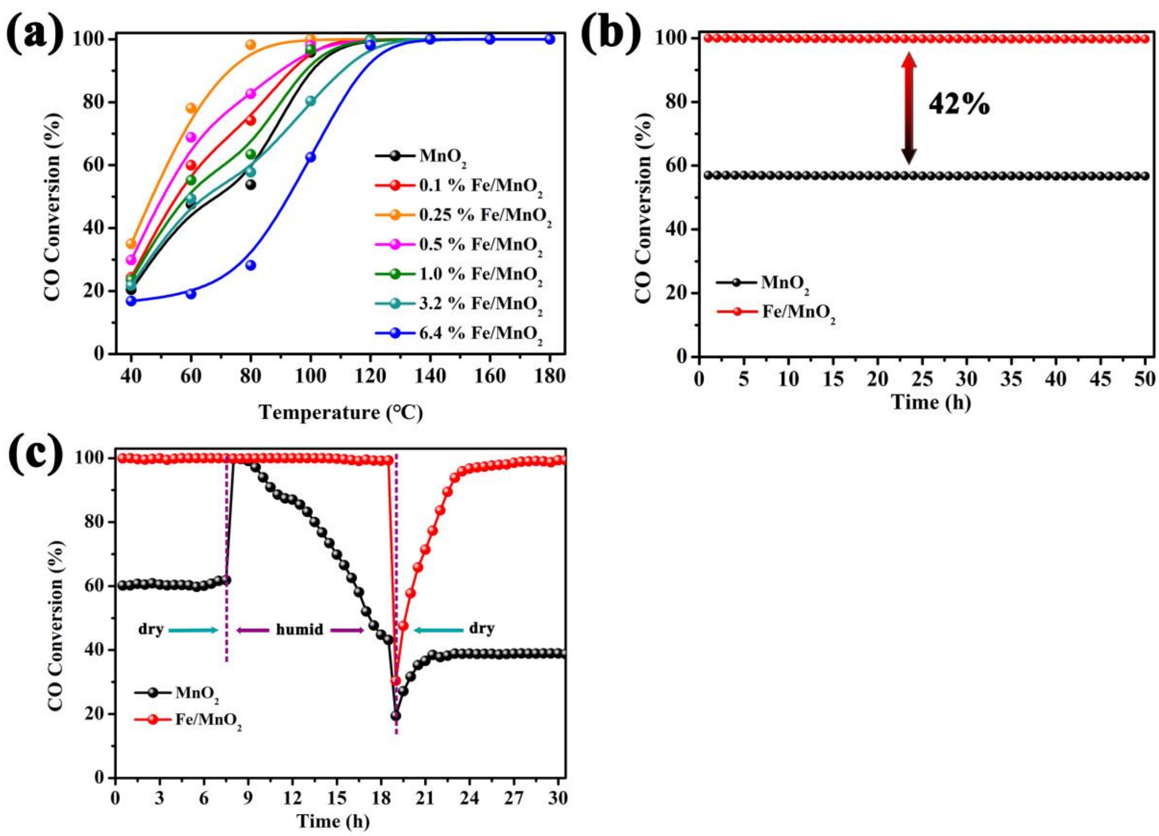

Supplementary Figure 7. (a) Light-off curves for CO oxidation of different proportions of Fe/MnO_2 and MnO_2. (b) The stability of catalysts in 50 h. (c) The tolerance test of water vapor for Fe/MnO_2 and MnO_2. (Reaction condition: 1% CO, 4% O_2, and N_2 as balanced gas, total flow rate 50 mL min^{-1} in a and b. Besides that, 1% H_2O is contained in the reaction system in c).

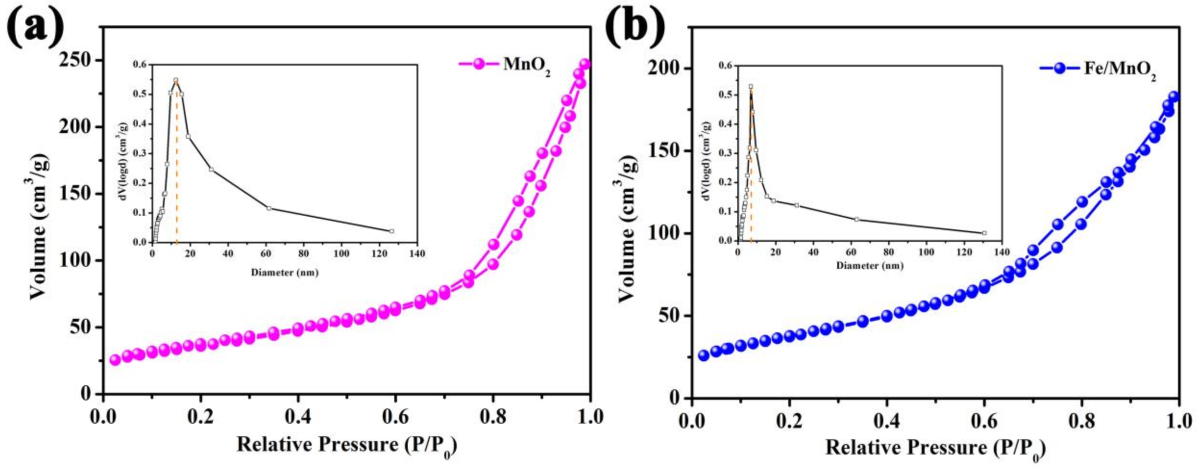

Supplementary Figure 8. Nitrogen adsorption/desorption isotherm plots of (a) MnO_2 and (b) Fe/MnO_2 (the insets of a and b showed the pore size distribution).

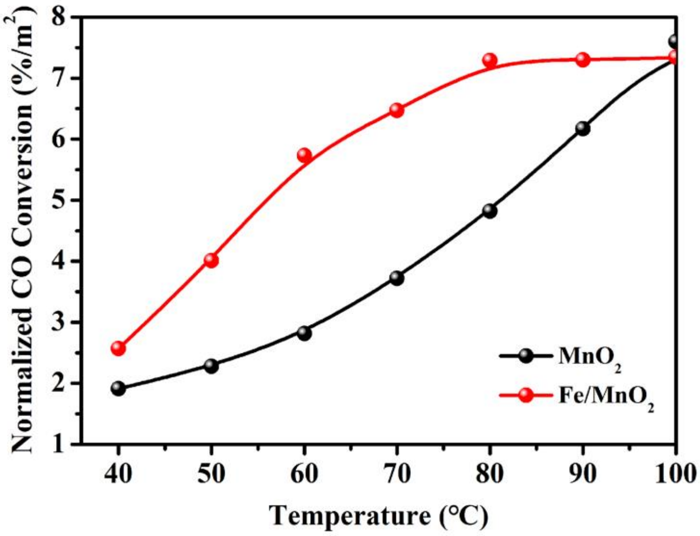

Supplementary Figure 9. CO catalytic performance of the MnO_2 and Fe/MnO_2 normalized by BET surface area recorded in Table S5.

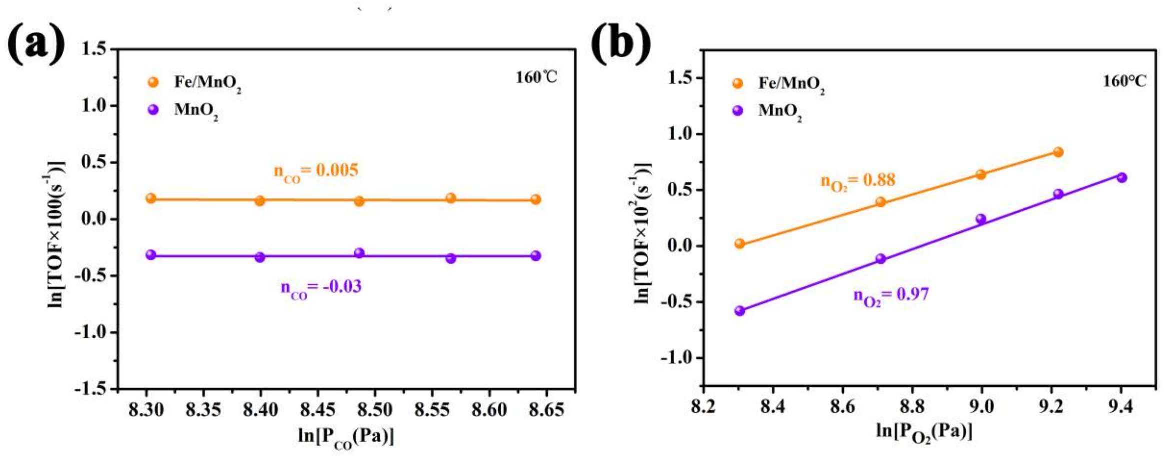

Supplementary Figure 10. Effect of (a) CO and (b) O_2 partial pressure on the TOF on supported Fe/MnO_2 and MnO_2 at 160 °C. P_{O_2} = 4 kPa in a and P_{CO}=4 kPa in b.

At low temperatures, Fe/MnO_2 was easier to activate molecular oxygen than MnO_2, which should be the essence for the better catalytic activity of Fe/MnO_2. Relatively, the reaction orders of O_2 increased at the higher temperature of 160 °C, and the gap between Fe/MnO_2 and MnO_2 reduced significantly. More surface-active sites were involved in the reaction, masking the promoting effect of Fe.

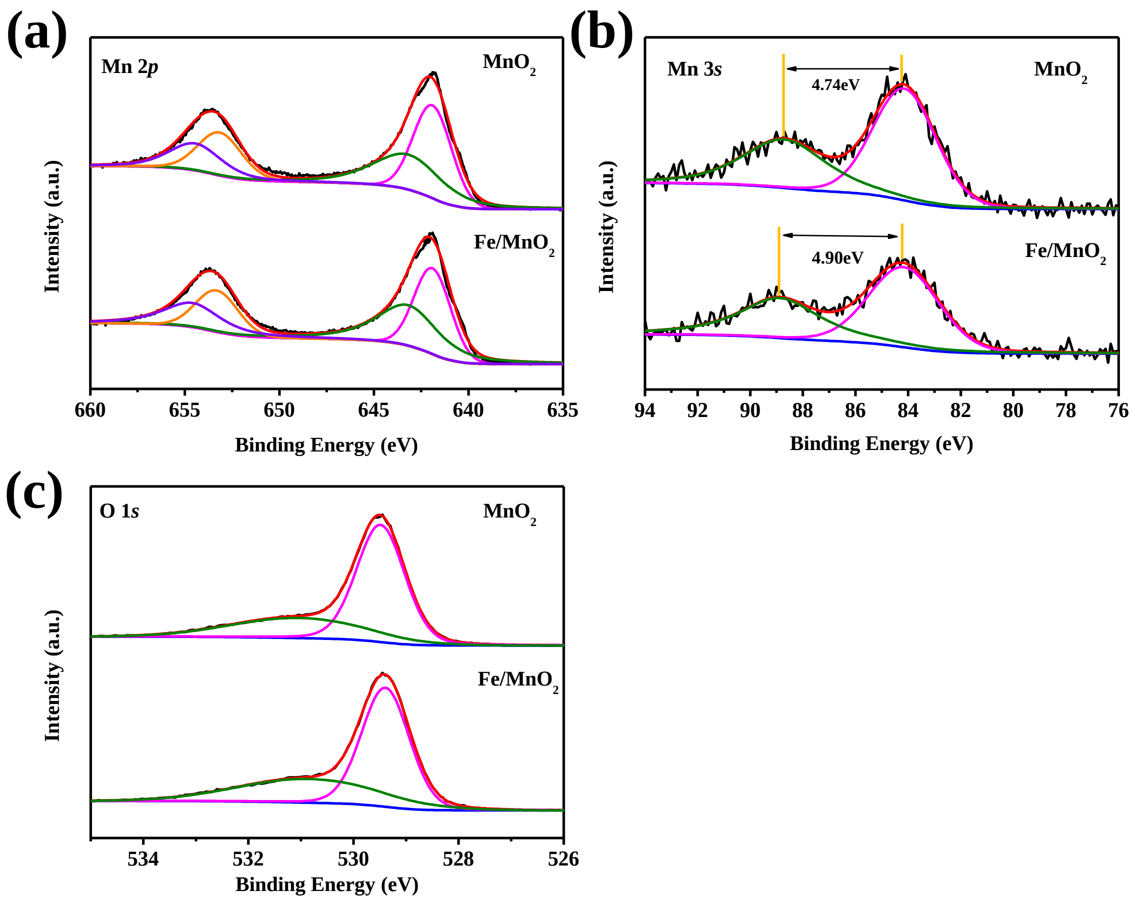

Supplementary Figure 11. (a) Mn 2*p*, (b) Mn 3*s*, and (c) O 1*s* XPS spectra of Fe/MnO_2 and MnO_2.

The XPS spectra of O 1*s* shown in Figure S11c was deconvoluted into two peaks at 531 and 529 eV, which were attributed to adsorbed oxygen species (including surface hydroxyl and surface adsorbed O_2) and lattice oxygen species, respectively [3]. Fe/MnO_2 owned a higher concentration of adsorbed oxygen species (37%) than MnO_2 (31%), indicating that it possessed a greater number of active sites, which will immensely promote the catalytic reaction of CO.

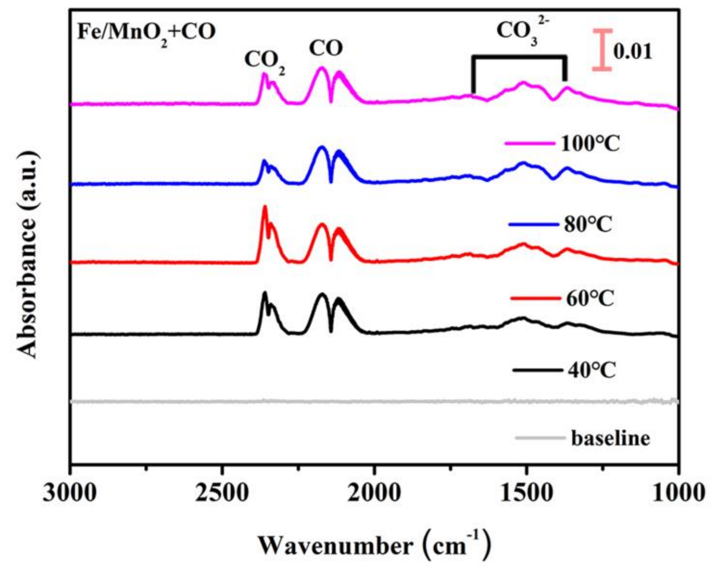

Supplementary Figure 12. In suit DRIFTS over Fe/MnO_2 in a continuous flow of 1% CO/4% O_2/N_2 in different temperature.

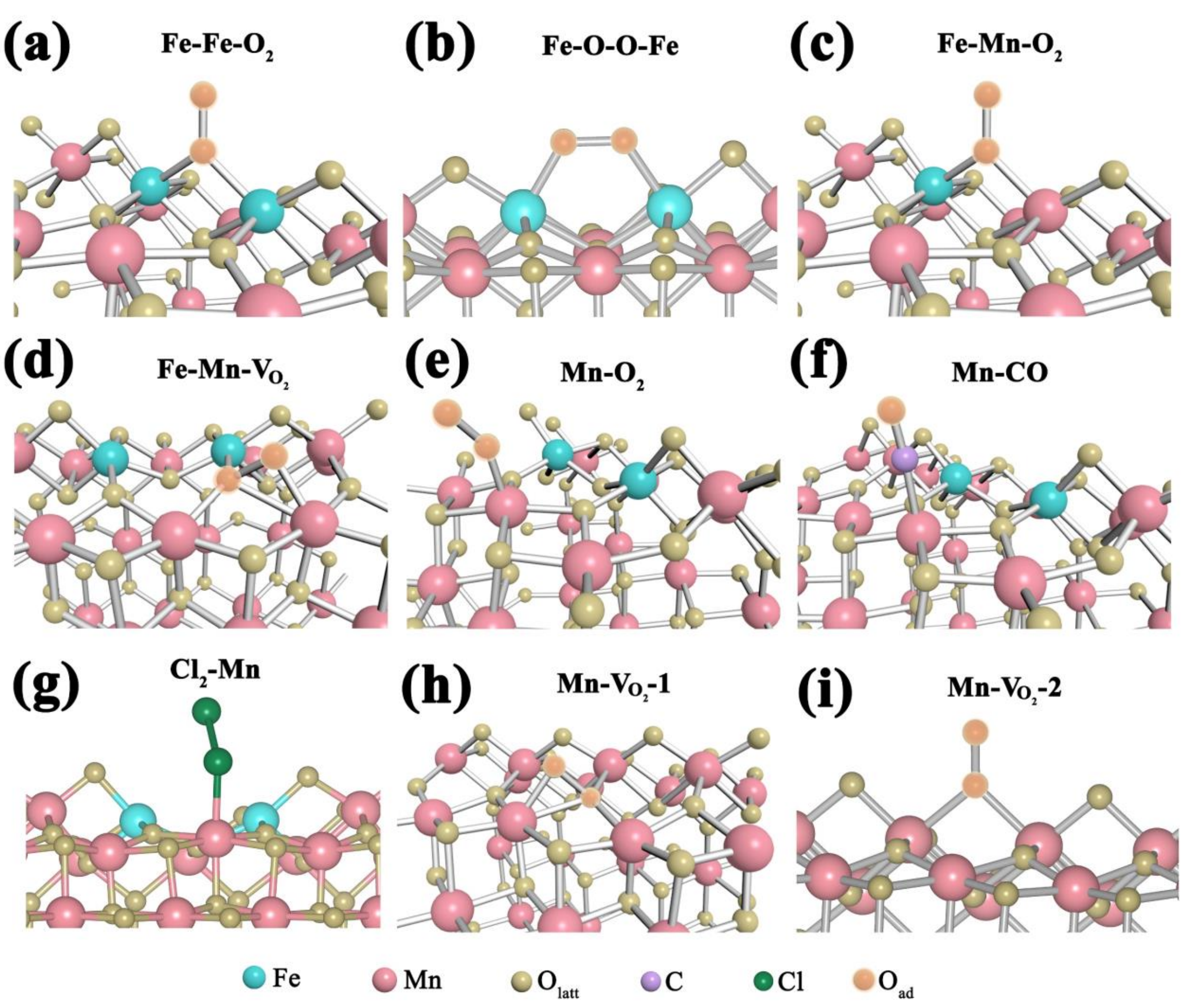

Configuration	a	b	c	d	e	f	g	h	i
Adsorption Energy (eV)	-2.00	-1.47	-1.82	-0.83	-1.66	-2.15	-3.11	-2.39	-1.69

Supplementary Figure 13. The stable configurations of (a-e) O_2, (f) CO and (g) Cl_2 adsorbed at the different sites of Fe/MnO_2 surface, and O_2 adsorbed in (h) the oxygen vacancy or (i) bi-manganese sites of MnO_2. The corresponding adsorption energy with zero-point energy correction is listed in the table of the picture.

Compared with O_2 linked to Mn site (-1.66 eV), CO will achieve preferential adsorption (-2.15 eV) under the reaction atmosphere conditions (Supplementary Fig. 13e, f). This will be further used in the later calculation of proposed reaction mechanism.

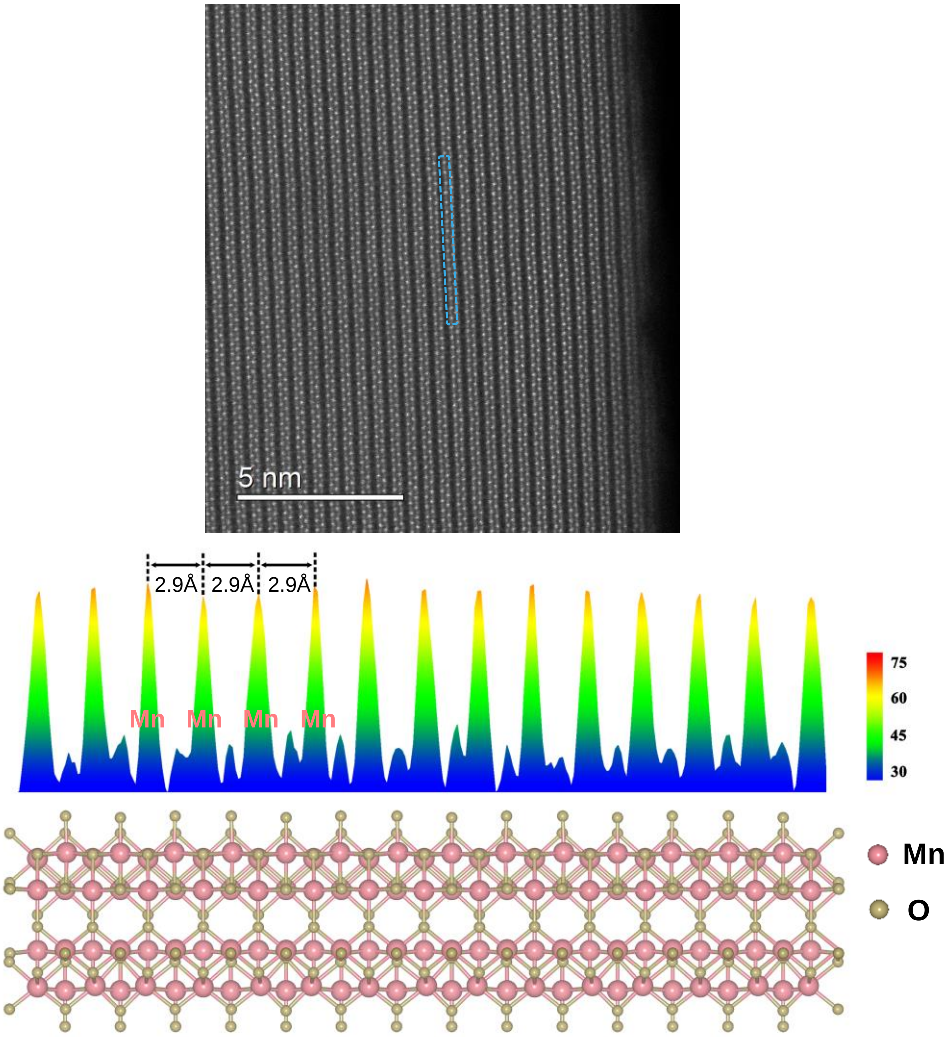

Supplementary Figure 14. STEM image of MnO_2 (top), intensity surface plot from blue dashed rectangle (middle) and the corresponding structural model (bottom).

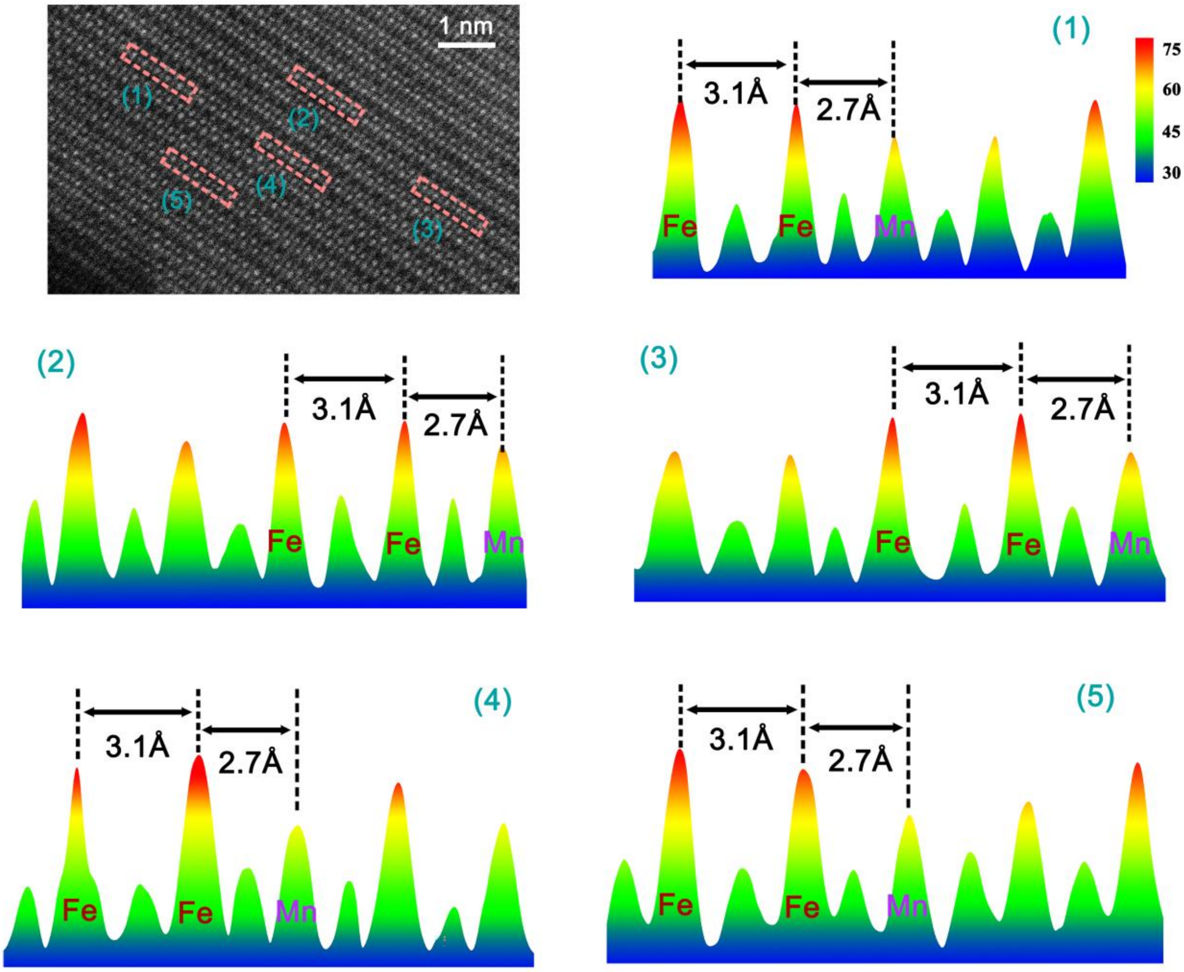

Supplementary Figure 15. Intensity surface plots in different position from STEM image of Fe/MnO_2.

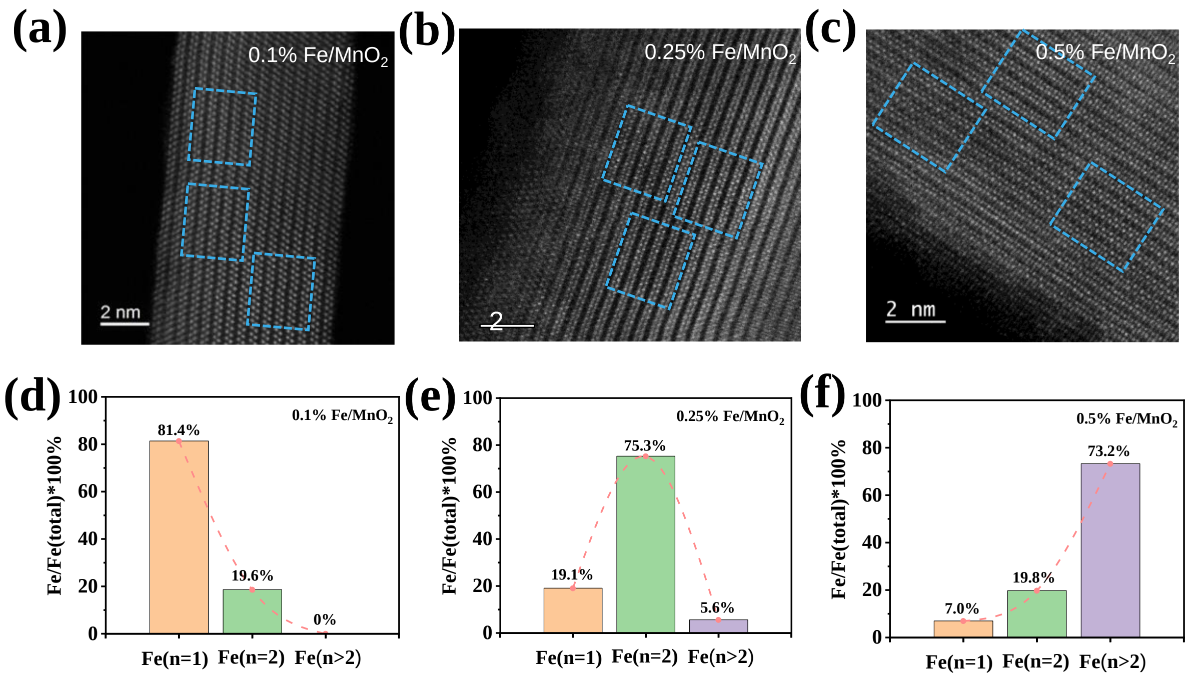

Supplementary Figure 16. (a-c) STEM images of different Fe/MnO_2 catalysts. The atoms surrounded by the blue dotted box will be used for site statistics. (d-f) Statistical results of the number of Fe sites corresponding to different catalysts.

We compared the activities of catalysts with different Fe introduction (0.1%, 0.25% and 0.5% Fe/MnO_2), and found that 0.25% Fe/MnO_2 exhibited the highest catalytic activity (Supplementary Fig. 7a). According to the statistical analysis of iron atoms distribution on the surface of three catalysts (Supplementary Fig. 16 and Supplementary Table 2), 0.1% Fe/MnO_2 possessed 81.4% of monatomic Fe, while 0.25% Fe/MnO_2 was of 75.3% two adjacent single-atom Fe sites. When the content of Fe reached 0.5%, the adjacent Fe sites ($n > 2$) became dominant (73.2%). Obviously, the catalytic activity of Fe/MnO_2 strongly depends on the surrounding environment of iron atoms and their interaction with MnO_2 support.

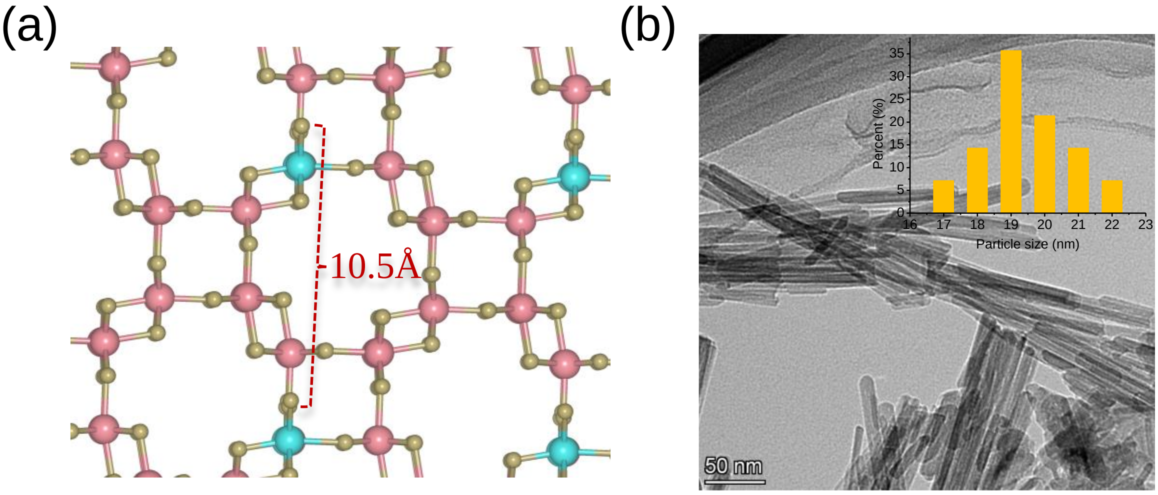

Supplementary Figure 17. (a) The structural model and (b) TEM spectrum of Fe/MnO_2. Particle size was counted according to TEM result.

Based on the strength of atoms and the distance between adjacent atoms, we statistically analyzed the STEM spectra of 300 atoms in three regions on the surface of three different Fe/MnO_2 catalysts (0.1%, 0.25% and 0.5% Fe/MnO_2) in Supplementary Fig. 16 and Supplementary Table 2. For 0.25% Fe/MnO_2, 89 of 300 atoms on the surface were counted as Fe atoms, and others as Mn atoms. According to the model structure of Fe/MnO_2 (Supplementary Fig. 17), the height of the monolayer atoms is 2.63 Å (h). The TEM results displayed that the thickness of the nanorods was about 19.7 nm (H), consistent with the literature [4]. On the basis of calculation formulas (5-9), the mass fraction of Fe was 0.46%, very close to the ICP test results (0.3%), if the impurities adsorbed on the surface were ignored. These results have demonstrated that Fe is distributed as a single atom on the surface of Fe/MnO_2, and it is feasible to distinguish Mn and Fe atoms according to the differences in strength and distance.

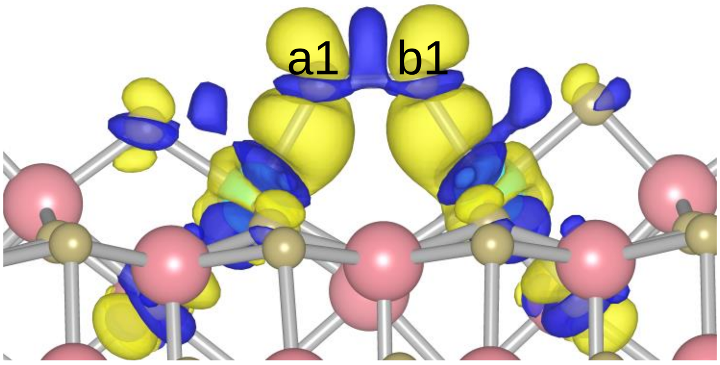

Oxygen atom	a1	b1
Bader Charge	-0.12	-0.12

Supplementary Figure 18. The difference charge density of O_2 adsorbed on Fe/MnO_2. Different oxygen atoms from adsorbed oxygen species were labeled as a1 and b1, and corresponding Bader charge was recorded in the bottom of charge density map. The charge density of yellow and blue represents the concentrated and scarce electrostatic potential scale respectively.

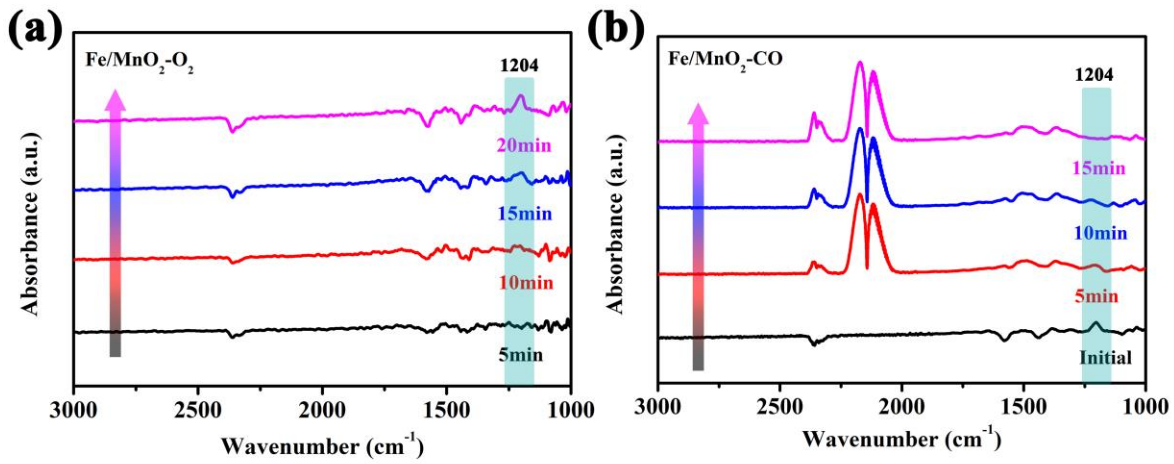

Supplementary Figure 19. In suit DRIFTS over Fe/MnO_2 in a continuous flow of (a) 4% O_2/N_2 and (b) 1% CO/N_2 over time at 40 ^oC.

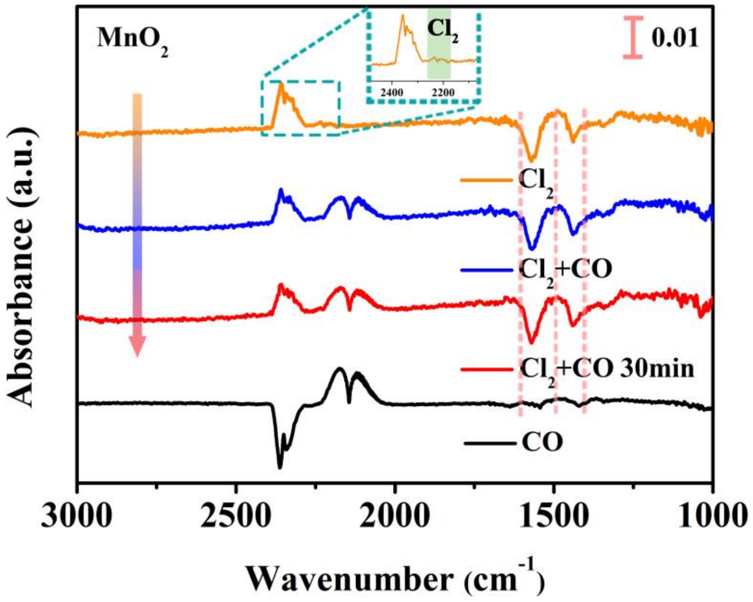

Supplementary Figure 20. In suit DRIFTS of MnO_2 with injection of Cl_2 in closed reactor at 40 ^oC, followed by CO and maintained for 30 min. The bottom line as a reference is recorded in the CO atmosphere. The inset shows partially enlarged spectra in Cl_2 atmosphere.

Chlorine is well known for its high electron density and strong adsorption properties. Some literatures reported that chlorine was prone to form strong adsorption with metal sites on the catalyst surface during the catalytic reaction, leading to catalyst deactivation [5,6]. Even after oxygen adsorption, Cl_2 could cover the surface of MnO_2 and firmly occupied the adsorption sites of CO. Furthermore, Cl_2 has a stronger adsorption capacity at unsaturated Mn sites than CO (-3.11 eV for Cl_2 versus -2.15 eV for CO), accounting for the design idea of the experiment. Therefore, we used Cl_2 to study the reaction mechanism in this study. After Cl_2 was injected into a reactor with MnO_2, characteristic peaks at 2270 and 2237 cm^{-1} appeared, which could be attributed to adsorption peaks of Cl_2 (the inset of Supplementary Fig. 20), indicating a strong interaction between Cl_2 and the MnO_2 surface. Subsequently, CO was injected into the closed system, the spectra has barely changed through 30 min, demonstrating that Cl_2 took priority in the adsorption site of CO and prevented the further occurrence of oxidation reaction.

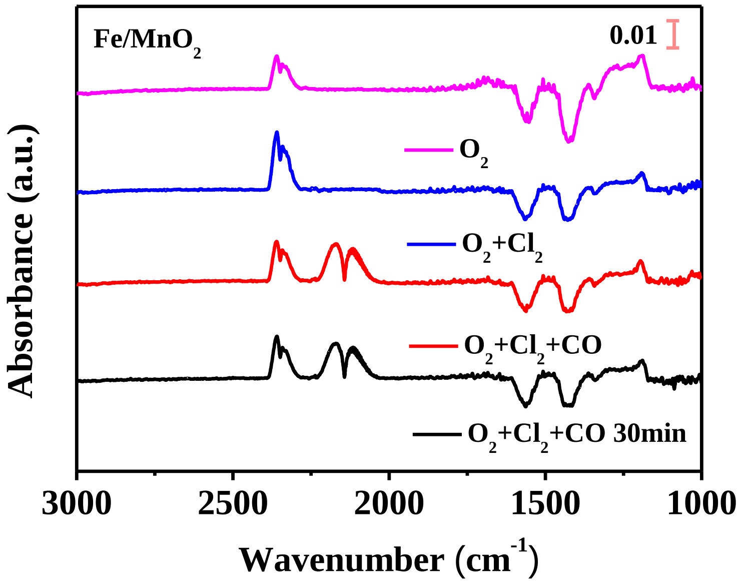

Supplementary Figure 21. In situ DRIFTS of MnO_2 with injection of O_2 in closed reactor at 100 °C, followed by Cl_2 at 40 °C, then CO at 40 °C, and finally maintained for 30 min.

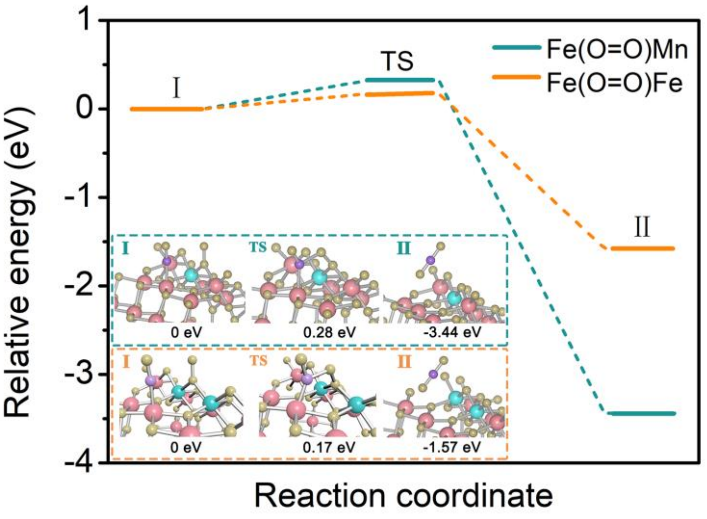

Supplementary Figure 22. Energy profiles of CO reacted with active species Fe(O=O)Mn and Fe(O=O)Fe. The optimized structures of initial states (I), transition states (TS) and final states (II) are listed in the dotted box, and the relative energy is recorded below the corresponding structure.

We compared the activation behavior of oxygen at Fe-Mn bimetallic sites. Relatively, the adsorption capacity of O_2 is weak at the double-center line composed of Fe and Mn (-1.82 eV vursus -2.00 eV for double Fe sites, Supplementary Fig. 13c), which may be the result of the less feedback π electrons transfered to oxygen from Mn d orbital. Refering to the energy profile of CO reacted with oxygen species at different active sites, remarkably, Fe(O=O)Fe performs lower potential barrier (0.17 eV) compared with Fe(O=O)Mn (0.28 eV). Moreover, final state of Fe(O=O)Fe has higher energy, corresponding to less stability, which is considered to facilitate the further migration of residual oxygen atoms (part V to VI in Fig. 5). Thus, the double Fe sites possess dominant ability to activate molecular oxygen.

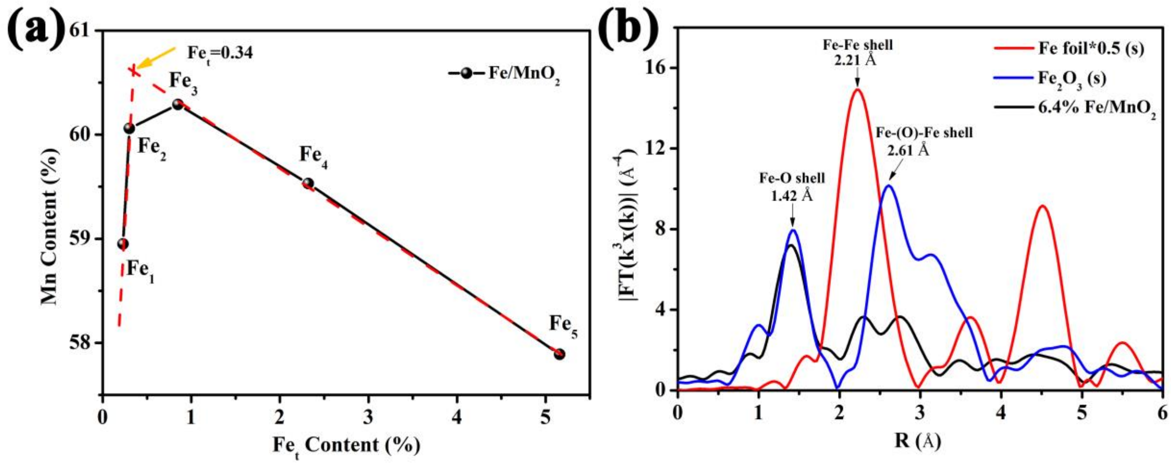

Supplementary Figure 23. (a) The relationship from the contents of Fe and Mn based on the result of ICP-OES. (b) The Fourier-transformed k-edge EXAFS spectra in R-space of Fe foil, Fe_2O_3 and 6.4% Fe/MnO_2.

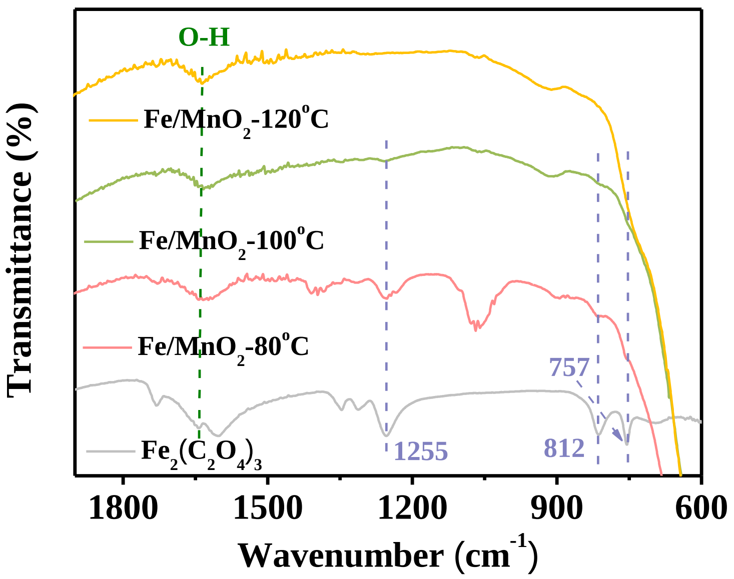

Supplementary Figure 24. ATR spectra of standard $Fe_2(C_2O_4)_3$ and Fe/MnO_2 synthesized from at different temperatures.

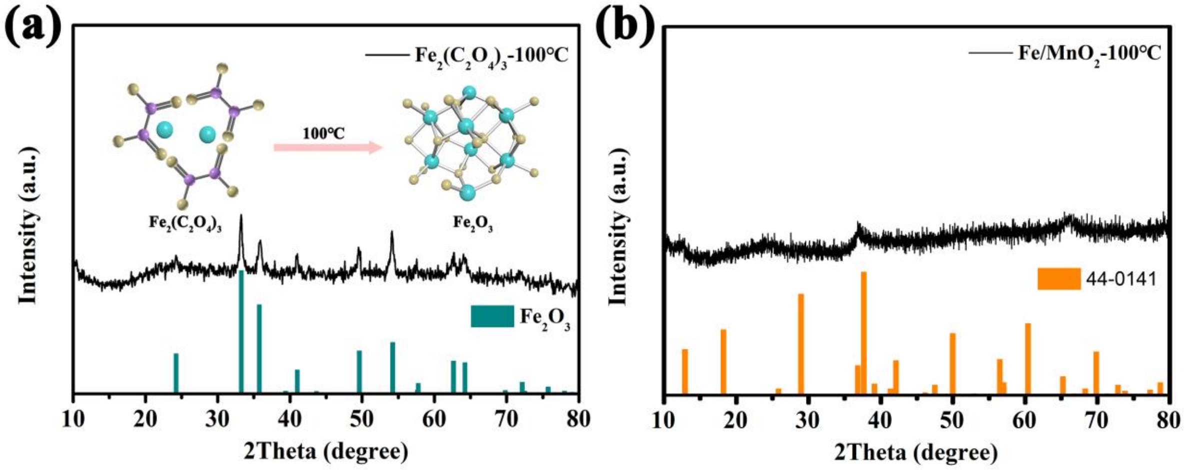

Supplementary Figure 25. XRD patterns of (a) $Fe_2(C_2O_4)_3$ and (b) Fe/MnO_2 prepared at 100 °C. (The inset shows the structural models of $Fe_2(C_2O_4)_3$ and Fe_2O_3 in a).

We conducted ATR measurements of Fe/MnO_2 synthesized at different temperatures (Supplementary Fig. 24). The characteristic peaks of ferric oxalate (757, 812 and 1255 cm^{-1}) appeared in the catalysts synthesized at 80 °C, which were weakened along with the further increase of the synthesis temperatures. In combination with Supplementary Fig. 25 and 26, the iron species existed in the precursor solution with the form of iron oxalate complex. With the increase of hydrothermal temperature, [MnO_6] structural units appeared in priority, resulting in the formation of the periodic structure of MnO_2. Then, the ferric oxalate was gradually decomposed into CO_2, leaving Fe atoms on the surface of MnO_2.

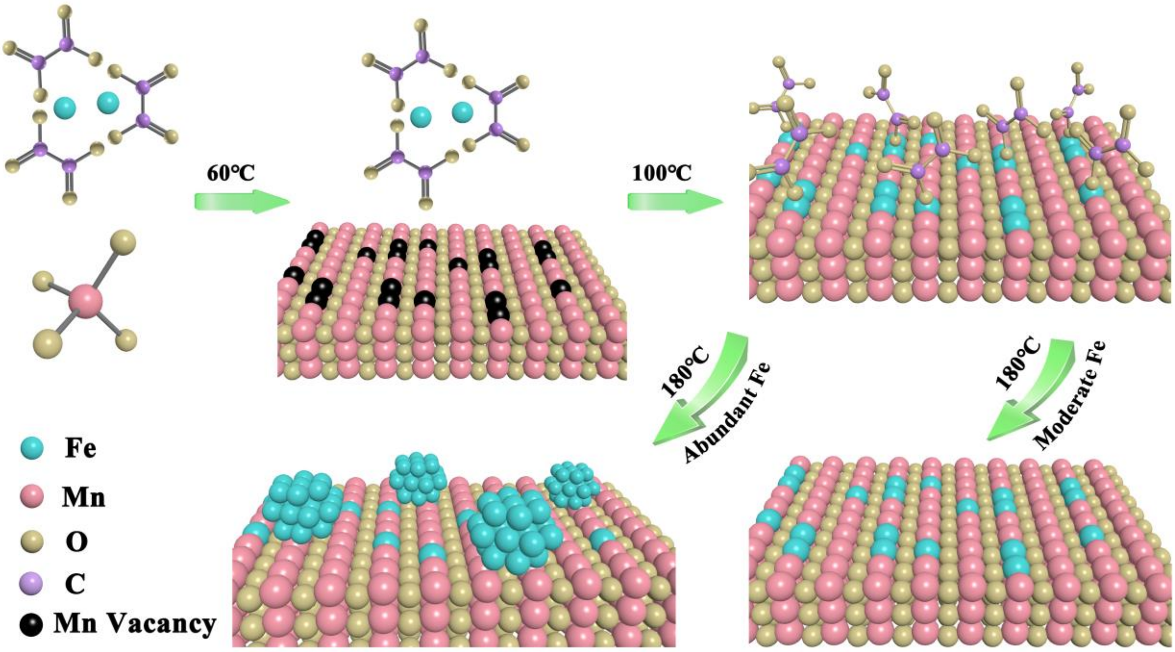

Supplementary Figure 26. Schematic illustration of growth mechanism of Fe/MnO_2.

ICP-OES of Fe/MnO_2 systems with different Fe contents were characterized, and the relative contents of Mn and Fe were recorded in Table S6. Interestingly, with increase of actual content of Fe, the relative content of Mn increased ay first and then decreased. The reasons for the change trend of Mn was discussed on the basis of formula:

$$C_{Mn} = \frac{m_{Mn}}{m_{Mn} + m_{Fe} + m_{other}} \quad (6)$$

(1) when the content of Fe was less than 1%, the introduction of Fe leaded to a sharp decrease in the content of other elements (m_{other}), including the content of lattice oxygen or the content of adsorbed species on the surface, resulting in the continuous increase in the relative content of Mn; (2) with the further increase of Fe content, Fe clusters were formed and covered on the catalyst surface, leading to the continuous decreased over the relative content of Mn. Then, the relative relationship between Mn and Fe content was used to discuss in Supplementary Fig. 23a, and the contents from Fe_3 to Fe_5 showed a good linear relationship. Combined with previous EXAFS analysis results, Fe existed as a single atom in Fe_2 catalyst, while Fe clusters appeared in Fe_5 catalyst (Supplementary Fig. 23b). Corresponding to the junction point of fitted curve for fe_1-fe_2 and fe_3-fe_5, the Fe content was 0.34%, which may be the

maximum for Fe existed only as a single atom. Therefore, by comparing the catalytic activity of catalysts with different Fe contents, the catalysts with 0.25% showed the optimal catalytic activity, which was attributed to the existence state of the monatomic form of Fe.

Based on above experimental results, the growth process of the catalyst as exhibited in Supplementary Fig. 26 maybe went through the following stages: (1) the precursor solution contained MnO_4^-, $C_2O_4^{2-}$, Fe^{3+} and so on. During the stirring process, $C_2O_4^{2-}$ was prone to form a chelate with Fe^{3+}, which existed in the form of $Fe_2(C_2O_4)_3$. (2) with the increase of temperature, MnO_4^- in the reduction of $C_2O_4^{2-}$ continued to generate the defect-rich skeleton of MnO_2, while $Fe_2(C_2O_4)_3$ remained stable in the solution. (3) when the temperature rose to 100 ^oC, $Fe_2(C_2O_4)_3$ began partial decomposition (shown in Supplementary Fig. 25). Under the action of $C_2O_4^{2-}$ unsaturated coordination, Fe atom kept getting closer to the MnO_2 surface with abundant defects, and then occupied the defect sites of Mn. (4) when the temperature reached the final 180 ^oC, $Fe_2(C_2O_4)_3$ was completely decomposed, and Fe existed as a single atom on the catalyst surface. On the other hand, if the amount of Fe atom far exceeds the number of defect sites on the catalyst surface, with the decomposition of $Fe_2(C_2O_4)_3$, the excess Fe atoms would continuously gather into Fe clusters and attach to the catalyst surface, thus impeding the adsorption of reactants on the catalyst surface and inhibiting the further reaction. Therefore, $C_2O_4^{2-}$ acted as a ligand to disperse Fe atomically, and the chelation ensured Fe dispersed on the surface of MnO_2, achieving the maximum utilization of Fe atom.

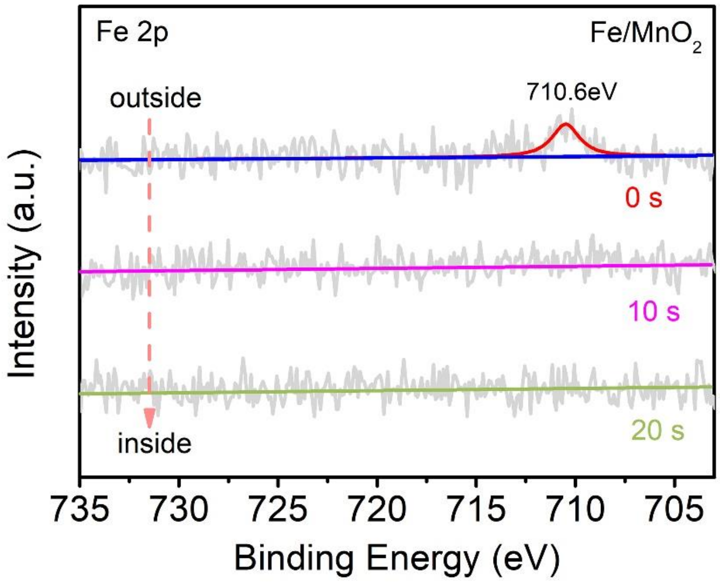

Supplementary Figure 27. Etching XPS spectra of Fe 2*p* in Fe/MnO_2 sample.

The characteristic peak of 710.6 eV corresponding to Fe species was detected on the surface of the catalyst by the etching XPS spectra. With the increase of etching time, the characteristic peak disappeared, indicating that Fe was distributed on the surface of the catalyst, which was consistent with the experimental results.

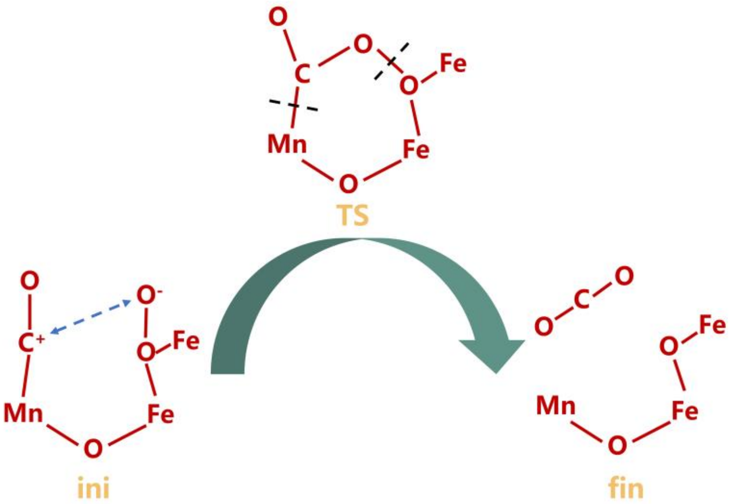

Supplementary Figure 28. Schematic diagram of Fe(O=O)Fe participating in CO oxidation from II→TS1→III in Fig. 5.

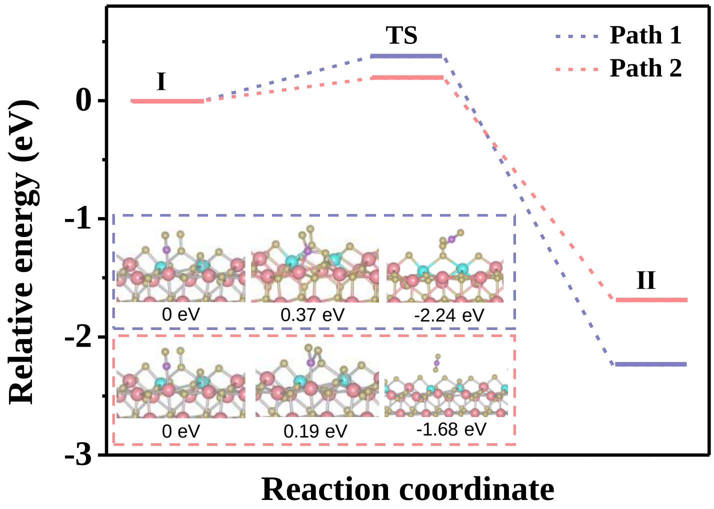

Supplementary Figure 29. Energy profiles of CO reacted through the different path in Fe/MnO_2. The optimized structures of initial states (I), transition states (TS) and final states (II) are listed in the dotted box, and the relative energy is recorded below the corresponding structure.

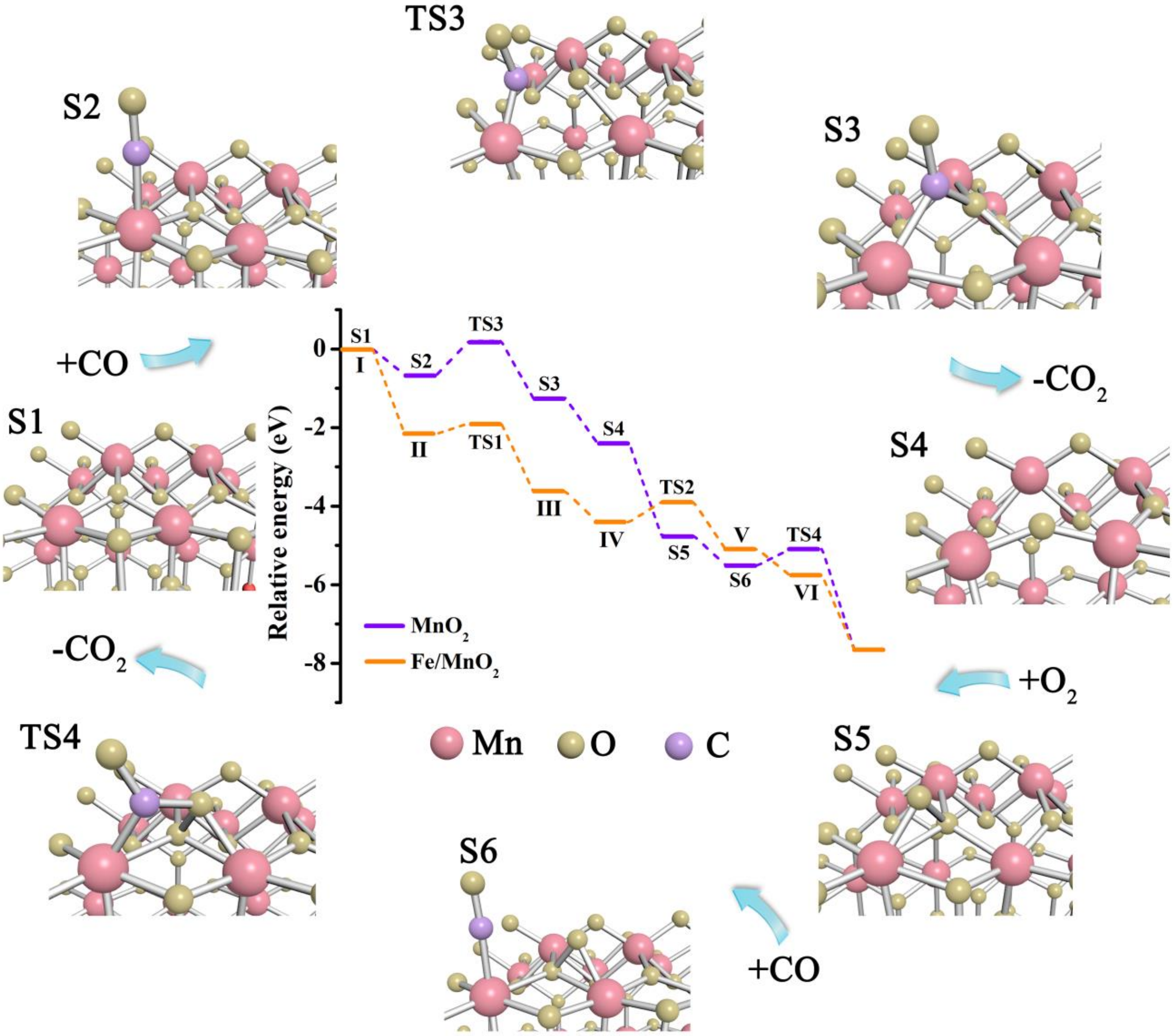

Supplementary Figure 30. Proposed reaction mechanism for CO oxidation on MnO_2 surface. The inset shows the calculated energy profiles in eV. The structures of intermediates and transition states are shown in the reaction cycle. The energy profile of the reaction cycle for Fe/MnO_2 is shown by the orange line in the inset (reaction cycle is shown in Fig. 5). The purple line shows the energy profile for MnO_2.

In this reaction, CO adsorbed at the Mn site first reacted with the adjacent lattice oxygen to form CO_2 and leave V_O. The surface V_O was refilled with oxygen molecules, which reacted with another CO molecule after that. After the desorption of CO_2, the catalyst returned to its original state.

Supplementary Tables

Supplementary Table 1. Surface chemical composition of MnO_2 and Fe/MnO_2 determined by XPS.

Catalysts	Mn^{3+}	Mn^{4+}	O_{ad}[a]	O_{latt}[b]	ΔE[c]	AOS[d]
MnO_2	42%	58%	31%	69%	4.74	3.62
Fe/MnO_2	53%	47%	37%	63%	4.90	3.44

a. The adsorbed oxygen species on catalyst surface.

b. The lattice oxygen species on catalyst surface.

c. The binding energy difference (ΔE) according to Mn 3*s* in XPS.

d. The average oxidation state. AOS of Mn was calculated according to the binding energy difference (ΔE) through an empirical formula: AOS = 8.956−1.126 × ΔE.

Supplementary Table 2. Statistical results of different Fe sites and Mn contents of the three catalysts from Supplementary Fig. 16.

	Fe(total)	Fe(n=1)	Fe(n=2)	Fe(n>2)	Mn
0.1% Fe/MnO_2	43	35	8	0	257
0.25% Fe/MnO_2	89	17	67	5	211
0.5% Fe/MnO_2	172	12	34	126	128

n represents the number of adjacent Fe atoms.

Supplementary Table 3. Fe k-edge EXAFS fitting parameters of Fe/MnO_2 for Fig. 1e.

Sample	Shell	C.N.	R (Å)	σ^2 (10^{-3} Å)	ΔE_0 (eV)
Fe/MnO_2	$Fe\text{-}O_1$	0.9±0.2	1.73±0.03	5.3±3.5	-8.3±0.2
	$Fe\text{-}O_2$	4.6±0.2	1.85±0.03	5.3±3.5	-8.3±0.2

C.N., σ_2, R, and ΔE_0 are the coordination number, interatomic distance, Debye-Waller factor, and shift in the edge energy.

Supplementary Table 4. The measurement condition of apparent activation energy over MnO_2 and Fe/MnO_2 in different temperature areas.

Catalysts	Reaction conditions	Ea ($kJ\ mol^{-1}$)
MnO_2	50 mg; 2% CO+4% O_2+N_2 balance; GHSV = 120,000 $mL\ g^{-1}\ h^{-1}$	70.1 (R^2 = 0.9999)
Fe/MnO_2	50 mg; 2% CO+4% O_2+N_2 balance; GHSV = 120,000 $mL\ g^{-1}\ h^{-1}$	33.7 (R^2 = 0.9956)

Supplementary Table 5. The measurement results of the MnO_2 and Fe/MnO_2 catalysts from BET.

Catalysts	Surface area (m^2 g^{-1})	Pore volume (cm^3 g^{-1})	Pore diameter (nm)
MnO_2	126.1	0.378	12.3
Fe/MnO_2	136.2	0.280	6.8

Supplementary Table 6. The content of Fe, Mn on MnO_2 and Fe/MnO_2 with different percentage content determined by ICP-OES.

Catalysts	Number	Fe_r[a] (%)	Fe_t[b] (%)	Mn (%)
MnO_2	Fe_0	0	0	56.47
Fe/MnO_2	Fe_1	0.1	0.23	58.95
Fe/MnO_2	Fe_2	0.25	0.3	60.06
Fe/MnO_2	Fe_3	1	0.85	60.29
Fe/MnO_2	Fe_4	3.2	2.32	59.53
Fe/MnO_2	Fe_5	6.4	5.16	57.89

a. The theoretical content of Fe based on the calculation.

b. The actual content of Fe according to the results of ICP-OES.

Supplementary Table 7. The integrated O_2-TPD and H_2-TPR peak area normalized for Fe/MnO_2 and MnO_2.

From O_2-TPD measurements

Catalysts	FeOO	V_{O_2}	S-O_{latt} (Mn^{3+} or Fe^{3+})	S-O_{latt} (Mn^{4+})	B-O_{latt} (Mn^{4+})
	Percentage of desorption area (%)				
MnO_2	0	6.53	20.19	43.32	29.96
Fe/MnO_2	9.08	17.32	37.77	21.57	14.27

From H_2-TPR measurements

Catalysts	FeOO	V_{O_2}	Surface (Mn^{3+} or Fe^{3+})	Surface (Mn^{4+})	Bulk (Mn^{4+})
	Percentage of reduction area (%)				
MnO_2	0	17.31	11.50	15.68	55.52
Fe/MnO_2	12.12	8.94	11.53	12.77	54.64

Supplementary Table 8. Calculated energy and zero-point energy of the corresponding free and adsorbed molecules.

Configuration	Energy (eV)	Zero Point Energy (eV)	Corrected Energy (eV)
Fe-Fe-O_2	-545.52	0.08	-545.44
Fe-O-O-Fe	-544.97	0.06	-544.91
Fe-Mn-O_2	-545.25	0.07	-545.18
Fe-Mn-VO_2	-547.20	0.05	-547.15
Mn-O_2	-545.19	0.09	-545.10
Mn-CO	-551.88	0.12	-551.76
Mn-VO_2-1	-548.80	0.05	-548.75
Mn-VO_2-2	-548.13	0.08	-548.05
Cl_2-Mn	-540.66	0.01	-540.65
O_2	-8.84	0.10	-8.74
CO	-14.80	0.13	-14.67
Cl_2	-3.58	0.03	-3.55

Supplementary Table 9. Energy changes for CO oxidation over Fe/MnO_2 and MnO_2 surface via DFT calculation.

Fe/MnO_2		MnO_2	
Step in Fig. 5	Relative energy (eV)	Step in Supplementary Fig. 30	Relative energy (eV)
I	0	S1	0
II	-2.159	S2	-0.657
TS1	-1.989	TS3	0.203
III	-3.623	S3	-1.247
IV	-4.404	S4	-2.408
TS2	-3.894	S5	-4.749
VI	-5.105	S6	-5.505
VI	-5.769	TS4	-5.095
Final state	-7.654	Final state	-7.654

Supplementary References

1 Montemore, M. M., van Spronsen, M. A., Madix, R. J. & Friend, C. M. O_2 activation by metal surfaces: implications for bonding and reactivity on heterogeneous catalysts. *Chem. Rev.* **118**, 2816-2862 (2018).

2 Gan, T. et al. Electron donation of non-oxide supports boosts O_2 activation on nano-platinum catalysts. *Nat. Commun.* **12**, 2741 (2021).

3 R. D. L. Smith, C. Pasquini, S. Loos, P. Chernev, K. Klingan, P. Kubella, M. R. Mohammadi, D. Gonzalez-Flores, H. Dau, *Nat. Commun.* **8**, 2022 (2017).

4 Rong, S., Zhang, P., Liu, F. & Yang, Y. Engineering crystal facet of α-MnO_2 nanowire for highly efficient catalytic oxidation of carcinogenic airborne formaldehyde. *ACS Catal.* **8**, 3435-3446, (2018).

5 He, C. et al. Recent advances in the catalytic oxidation of volatile organic compounds: a review based on pollutant sorts and sources. *Chem. Rev.* **119**, 4471-4568 (2019).

6 Dai, Q. et al. HCl-tolerant H_xPO_4/RuO_x–CeO_2 catalysts for extremely efficient catalytic elimination of chlorinated VOCs. *Environ. Sci. Technol.* **55**, 4007-4016 (2021).
